# Multilocus sequence typing supports the hypothesis that *Ochrobactrum anthropi *displays a human-associated subpopulation

**DOI:** 10.1186/1471-2180-9-267

**Published:** 2009-12-18

**Authors:** Sara Romano, Fabien Aujoulat, Estelle Jumas-Bilak, Agnès Masnou, Jean-Luc Jeannot, Enevold Falsen, Hélène Marchandin, Corinne Teyssier

**Affiliations:** 1 Université Montpellier 1, Laboratoire de Bactériologie-Virologie, EA 3755 UM1, Faculté de Pharmacie, 15, Avenue Charles Flahault, BP 14491, 34093 Montpellier Cedex 5, France; 2 Culture Collection, University of Göteborg, Mikrobiologen, Guldhedsgatan 10, SE-413 46 Göteborg, Box 7193, SE-402 34 Göteborg, Sweden; 3 Centre Hospitalier Régional Universitaire de Montpellier, Hôpital Arnaud de Villeneuve, Laboratoire de Bactériologie, 371 Avenue du Doyen Gaston Giraud, 34295 Montpellier Cedex 5, France

## Abstract

**Background:**

*Ochrobactrum anthropi *is a versatile bacterial species with strains living in very diverse habitats. It is increasingly recognized as opportunistic pathogen in hospitalized patients. The population biology of the species particularly with regard to the characteristics of the human isolates is being investigated. To address this issue, we proposed a polyphasic approach consisting in Multi-Locus Sequence Typing (MLST), multi-locus phylogeny, genomic-based fingerprinting by pulsed-field gel electrophoresis (PFGE) and antibiotyping.

**Results:**

We tested a population of 70 *O. anthropi *clinical (n = 43) and environmental (n = 24) isolates as well as the type strain *O. anthropi *ATCC49188^T ^and 2 strains of *Ochrobactrum lupini *and *Ochrobactrum cytisi *isolated from plant nodules. A Multi-Locus Sequence Typing (MLST) scheme for *O. anthropi *is proposed here for the first time. It was based on 7 genes (3490 nucleotides) evolving mostly by neutral mutations. The MLST approach suggested an epidemic population structure. A major clonal complex corresponded to a human-associated lineage since it exclusively contained clinical isolates. Genomic fingerprinting separated isolates displaying the same sequence type but it did not detect a population structure that could be related to the origin of the strains. None of the molecular method allowed the definition of particular lineages associated to the host-bacteria relationship (carriage, colonisation or infection). Antibiotyping was the least discriminative method.

**Conclusion:**

The results reveal a human-associated subpopulation in our collection of strains. The emergence of this clonal complex was probably not driven by the antibiotic selective pressure. Therefore, we hypothesise that the versatile species *O. anthropi *could be considered as a human-specialized opportunistic pathogen.

## Background

*Ochrobactrum anthropi *is a highly versatile alphaproteobacterium with ability to colonize an exceptionally wide variety of habitats, from hostile environments such as polluted soil [1,2], to plants [2], nematodes [3], insects [4], animals [5] and man [6]. Two other species, *Ochrobactrum lupini *and *Ochrobactrum cytisi*, have been isolated from leguminosae nodules [7,8] and were genetically undistinguishable from *O. anthropi *[9,10]. The 10 other species of the genus *Ochrobactrum *[11] could be discriminated on the basis of 16S rDNA sequences but this marker was too conserved to allow a study of interrelationships among each species [9]. According to their habitat and/or to the relationships with their host, the population structure of *O. anthropi *varied. For example, biological and genomic microdiversity was higher in bulk soil than in the rhizosphere [12,13]. Authors related this difference in diversity level to the expansion of clones adapted to metabolites produced by rhizoredeposition [13]. Human clinical isolates of *O. anthropi *appeared diverse when analyzed by Pulsed Field Gel Electrophoresis (PFGE) [14], rep-PCR [13] and Internal Transcribed Spacer (ITS) sequencing [15].

Opportunistic infections and nosocomial outbreaks due to *O. anthropi *have been increasingly reported during the last decade, particularly in patients with indwelling devices [16], in dialysis [17] or after surgery [18]. *O. anthropi *was described as one of the Gram-negative rods most resistant to common antibiotics. It resists particularly to all β-lactams, except imipenem by production of an AmpC β-lactamase, OCH-1, described as chromosomal, inducible, and resistant to inhibition by clavulanic acid [19]. As the virulence of *O. anthropi *appeared to be low, its resistance to antimicrobial agents could be the major feature explaining its increasing role in human infectious diseases. However, some case reports suggested higher virulence for some strains, which are capable of producing pyogenic monomicrobial infections [20] or life-threatening infections such as endocarditis [21]. In addition, the genome of the type strain *O. anthropi *ATCC 49188^T ^has been recently sequenced and contains a complete homolog of the *virB *operon (accession number: CP000758) on the large chromosome of the bipartite genome. This operon is the major determinant of the virulence of alpha-proteobacteriarelated to the genus *Ochrobactrum*. In *Brucella *spp., it allows the intra-macrophagic survival and multiplication of the bacterium [22]. It is also the main support for DNA transfer and for phytopathogenicity in *Agrobacterium tumefaciens *[23]. In the case of opportunistic pathogens, which generally do not fully respond to Koch's postulate, the link between virulence-related genes and infection is not clearly established. For example, opportunistic *Escherichia coli *involved in bacteremia showed a different content of virulence genes between strains, and the distribution of the virulence-related genes was independent of the host [24]. In contrast, MLST based on sequences of housekeeping genes sequences provides evidence for positive correlation between virulence, invasiveness and clonal origin of the *E. coli *strains [24]. Therefore, the behaviour of opportunistic pathogens could be considered as a clonal adaptation to human ecology. There is evidence that sequence clusters for a given gene can correspond to ecologically distinct populations, even for genes not related to the adaptative divergence between populations [25,26].

We investigated the existence of human-adapted subpopulations of *O. anthropi *by the study of its population structure using multi-locus housekeeping genotyping. We used genomic fingerprinting by PFGE and antibiotyping to provide complementary data to support MLST interpretation.

## Methods

### Bacterial strains

The 70 strains studied were described in Table 1 and Table 2. Fourty-three strains were clinical isolates. Among them 33 were obtained from patients hospitalized in 5 French hospitals in Montpellier, Nîmes (south-eastern France), Clermont-Ferrand (central France), Nancy (north-eastern France) and Toulouse (south-central France) from 1998 to 2007 and in Denmark. Ten collection strains isolated from man in Europe and the United States were also included as well as *O. anthropi *ATCC 49188^T^. The isolates were representative of different host-bacteria relationships, carriage or colonization without clinical symptoms or infection. Twenty-four environmental strains were from diverse origins, including water, soil, rhizophere, nematodes and industrial processes. The type strain of the species *O. cytisi *and a reference strain of *O. lupini *were also included. The affiliation of the isolates to *O. anthropi *was assessed as previously described [6]. Briefly, urease production and colistin, tobramicin and netilmicin susceptibility determined by disk diffusion assay gave presumptive identification of the species *O. anthropi*. The identification was confirmed by *rrs *(16S rDNA) [10] and *recA *sequencing [9].

**Table 1:** Characteristics of the *O. anthropi *strains of human origin.

Strains	MSCC	eBCC	ST	Allelic profiles							PFGE cluster	Origin	Region, country and year of isolation
							
				*dnaK*	*recA*	*rpoB*	*aroC*	*omp25*	*trpE*	*gap*			
**CCUG34461**	MSCC1	eBCC1	**1**	1	1	4	1	9	7	4	ND	Trachea	Tromsö, Norway, 1995

**CIP103948**	MSCC1	eBCC1	**1**	1	1	4	1	9	7	4	ND	Foot Wound	Fr, 1978

**ADV17**	MSCC11	eBCC1	**2**	1	4	7	6	1	7	4	I	Digestive tract (c)	Montpellier, Fr, 2001

**ADV23**	MSCC4	eBCC4	**3**	4	1	1	1	1	4	1	IV	Bone marrow (i)	Montpellier, Fr, 2002

**ADV34**	MSCC4	eBCC4	**3**	4	1	1	1	1	4	1	VI	Digestive tract (c)	Montpellier, Fr, 2003

**ADV77**	MSCC4	eBCC4	**3**	4	1	1	1	1	4	1	II	Digestive tract (c)	Montpellier, Fr, 2006

**ADV8**	MSCC4	eBCC4	**3**	4	1	1	1	1	4	1	I	Digestive tract (c)	Montpellier, Fr, 1999

**ADV97**	MSCC4	eBCC4	**3**	4	1	1	1	1	4	1	Vb	Digestive tract (c)	Montpellier, Fr, 2007

**NAN63**	MSCC4	eBCC4	**3**	4	1	1	1	1	4	1	II	Blood (i)	Nancy, Fr, 2005

**ADV29**	MSCC4	eBCC4	**4**	4	1	1	1	9	4	1	IV	Urine (i)	Montpellier, Fr, 2002

**ADV48**	MSCC4	eBCC4	**4**	4	1	1	1	9	4	1	III	Respiratory tract (c)	Montpellier, Fr, 2004

**ADV61**	MSCC4	eBCC4	**4**	4	1	1	1	9	4	1	IV	Respiratory tract (c)	Montpellier, Fr, 2005

**CCUG28303**	MSCC4	eBCC4	**4**	4	1	1	1	9	4	1	ND	Blood (i)	Göteborg, Sw, 1991

**LMG34**	MSCC4	eBCC4	**4**	4	1	1	1	9	4	1	ND	Pleural fluid (i)	Denmark, NA

**ADV38**	MSCC4	eBCC4	**5**	4	1	1	1	5	4	1	IV	Digestive tract (c)	Montpellier, Fr, 2003

**CCUG20020**	MSCC4	eBCC4	**5**	4	1	1	1	5	4	1	ND	Blood (i)	Göteborg, Sweden, 1991

**LMG5435**	MSCC4	eBCC4	**5**	4	1	1	1	5	4	1	ND	Blood (i)	UK, 1983

**ADV40**	S	S	**6**	5	4	1	11	6	1	7	VI	Digestive tract (c)	Montpellier, Fr, 2003

**ADV53**	MSCC4	eBCC4	**7**	4	1	1	9	9	4	1	IV	Throat (c)	Montpellier, Fr, 2004

**ADV99**	MSCC4	eBCC4	**7**	4	1	1	9	9	4	1	IV	Blood (i)	Montpellier, Fr, 2007

**ADV64**	MSCC4	eBCC4	**8**	4	1	1	8	7	4	1	VI	Wound (i)	Montpellier, Fr, 2005

**ADV72**	MSCC4	eBCC4	**9**	4	1	1	6	5	4	1	III	Digestive tract (c)	Montpellier, Fr, 2006

**ADV74**	S	eBCC21	**10**	5	1	3	3	8	11	9	III	Digestive tract (c)	Montpellier, Fr, 2006

**ADV75**	MSCC11	eBCC1	**11**	1	4	7	1	6	7	4	IV	Respiratory tract (c)	Montpellier, Fr, 2006

**ADV79**	S	eBCC1	**12**	1	4	7	10	6	7	4	I	Respiratory tract (c)	Montpellier, Fr, 2006

**ADV88**	MSCC1	eBCC1	**13**	1	1	4	1	1	6	4	II	Digestive tract (c)	Montpellier, Fr, 2007

**ADV90**	S	S	**14**	4	1	2	9	9	12	10	IV	Digestive tract (c)	Montpellier, Fr, 2007

**ADV91**	MSCC4	eBCC4	**15**	4	1	9	1	1	5	1	Vb	Digestive tract (c)	Montpellier, Fr, 2007

**CCUG33786**	MSCC4	eBCC4	**15**	4	1	9	1	1	5	1	ND	Blood (i)	Jönköping, Sw, 1995

**ADV92**	MSCC4	eBCC4	**16**	4	1	1	1	6	4	1	I	Respiratory tract (i)	Montpellier, Fr, 2007

**CCUG1235**	S	S	**22**	5	4	7	1	6	14	12	ND	Kidney transplant	Göteborg, Sw, 1971

**CLF18**	MSCC4	eBCC4	**23**	4	1	1	13	1	4	1	I	Throat (c)	Clermont-Ferrand, Fr, 1998

**CLF19**	S	S	**24**	5	4	7	11	1	1	12	III	Throat (c)	Clermont-Ferrand, Fr, 2000

**CLF20**	MSCC1	eBCC1	**25**	1	1	4	1	9	2	4	Va	Throat (c)	Clermont-Ferrand, Fr, 2000

**DNK118**	MSCC4	eBCC4	**26**	4	1	12	1	9	5	1	ND	Blood (i)	Denmark, 1996

**NIM27**	MSCC1	eBCC1	**32**	1	1	4	10	2	7	4	I	Blood (i)	Nîmes, Fr, 2002

**LMG3298**	MSCC4	eBCC4	**36**	4	1	1	15	9	4	1	ND	Blood (i)	Louisiana, USA, 1977

**LMG3303**	MSCC1	eBCC1	**37**	1	1	4	1	14	7	4	ND	Blood (i)	Fr, 1982

**NIM123**	MSCC11	eBCC1	**40**	1	4	7	11	6	7	4	ND	Eyes	Nîmes, Fr, 2002

**NIM28**	MSCC4	eBCC4	**41**	4	1	4	10	6	4	1	I	Wound (i)	Nîmes, Fr, 2002

**TOUL49**	S	S	**42**	1	1	7	9	10	7	4	Vb	Respiratory tract (i)	Toulouse, Fr, 2004

**TOUL58**	MSCC4	eBCC4	**43**	4	1	1	7	7	4	1	I	Respiratory tract (i)	Toulouse, Fr, 2004

**TOUL59**	S	S	**44**	5	3	4	9	5	1	8	VI	Respiratory tract (i)	Toulouse, Fr, 2004

**ATCC 49188**^**T**^	MSCC4	eBCC4	**4**	4	1	1	1	9	4	1	Va	Probably clinical	NA, before 1988

Minimum Spanning Clonal Complex (MSCC), eBURST clonal complex (eBCC), sequence type (ST) designation, allelic profiles and PFGE clusters at 60% similarity level. Life-style is indicated in the origin column: (i) for infection, (c) for colonization or carriage. S, singleton; ND, not determined; NA, not available, Fr, France, Sw, Sweden, UK, United Kingdom.

**Table 2:** Characteristics of the *O. anthropi *strains of environmental origin.

Strains	MSCC	eBCC	ST	Allelic profiles							PFGE cluster	Origin	Region, country and year of isolation
							
				*dnaK*	*recA*	*rpoB*	*aroC*	*omp25*	*trpE*	*gap*			
**11A**	MSCC1	eBCC1	**1**	1	1	4	1	9	7	4	Vb	Saline soil	Cordoba, Argentina, 2006

**DSM 2577**	MSCC1	eBCC1	**1**	1	1	4	1	9	7	4	II	Estuary sediments	Germany, 1983

**LMG 18953**	MSCC1	eBCC1	**1**	1	1	4	1	9	7	4	ND	Soil	Grignon, Fr, 1997

**LMG 2136**	MSCC1	eBCC1	**1**	1	1	4	1	9	7	4	Vb	Sewage plant waste water	Boras, Sweden, 1978

**PR38/sat**	MSCC1	eBCC1	**1**	1	1	4	1	9	7	4	Vb	*Heterorhabditis indica *(d)	Puerto Rico, 1996

**CCM 4352**	S	eBCC35	**17**	4	4	7	2	4	3	9	I	Pasteurized milk	Olomouc, Czech Republic, 1993

**CCM 999**	S	S	**18**	2	6	10	11	13	13	2	III	Arsenical cattle- dipping fluid	Queensland, Australia, 1960

**CCUG 18681**	MSCC1	eBCC1	**19**	1	1	4	10	6	7	4	II	Industrial dust	Göteborg, Sweden, 1986

**CCUG 32009**	MSCC1	eBCC1	**20**	1	1	4	2	3	7	4	VI	Paint	Boras, Sweden, 1993

**CCUG 54617**	S	eBCC21	**21**	5	1	3	2	4	11	9	IV	Industrial environment	Sweden, 2007

**NCCB 94107**	S	eBCC21	**21**	5	1	3	2	4	11	9	I	Marine sediments	Amsterdam, Nederland, 1994

**DSM 14396**	MSCC1	eBCC1	**27**	1	1	4	4	9	7	4	II	Agricultural soil	Germany, NA

**LMG 18952**	MSCC1	eBCC1	**27**	1	1	4	4	9	7	4	II	Wheat rhizoplane	Grignon, Fr, 1997

**DSM 20150**	S	S	**28**	3	2	8	12	12	10	3	I	Urine leech	Germany, NA

**FRG17/sat**	S	eBCC1	**29**	4	1	4	1	9	6	4	I	*Heterorhabditis indica *(d)	Guadeloupe, Fr, 1996

**FRG19/sat**	S	S	**30**	5	5	7	6	1	9	11	I	*Heterorhabditis indica *(d)	Guadeloupe, Fr, 1996

**ITHC13-3**	S	eBCC31	**31**	5	4	5	10	4	9	5	III	*Heterorhabditis indica *(d)	Italy, 2007

**ITHLA3-3**	MSCC1	eBCC1	**32**	1	1	4	10	2	7	4	I	*Heterorhabditis indica *(d)	Italy, 2007

**LR1**	MSCC1	eBCC1	**32**	1	1	4	10	2	7	4	I	Domestic water	Montpellier, Fr, 2004

**LR2**	MSCC1	eBCC1	**32**	1	1	4	10	2	7	4	I	Domestic water	Montpellier, Fr, 2004

**LMG 2133**	MSCC33	eBCC31	**33**	5	4	7	10	4	9	5	Va	Chromatography gel	Göteborg, Sweden, 1981

**LMG 7991**	S	eBCC35	**35**	4	4	7	10	4	3	9	I	Denifrication reactor	Belgium, 1979

**NCCB 90045**	S	S	**39**	5	4	7	1	9	9	7	ND	Activated sludge	Oosterschelde, Nederland, NA

**VAL**	MSCC33	eBCC31	**38**	5	4	6	5	4	9	5	III	*Heterorhabditis indica *(d)	Valescure, Fr, 2006

***O. cytisi *LMG 22713**^**T**^	S	S	**34**	6	4	11	14	11	8	6	Vb	*Cytisus scoparius *nodules (s)	Sevilla, Spain, 2002

***O. lupine *LMG 22727**	S	eBCC35	**35**	4	4	7	10	4	3	9	I	*Lupinus honoratus *nodules (s)	Cordoba, Argentina, 2002

Isolation data, Minimum Spanning Clonal Complex (MSCC), eBURST clonal complex (eBCC), sequence type (ST) designation, allelic profiles and PFGE clusters at 60% similarity level. Life-style is indicated in the origin column: (d) for dixeny and (s) for symbiosis. S, singleton; ND, not determined; NA, not available; Fr, France.

### Antibiotyping

The antimicrobial susceptibility profile was determined by the disk-diffusion assay on Mueller-Hinton (MH) agar and interpreted according to the guidelines of the Comité de l'Antibiogramme de la Société Française de Microbiologie [27]. Antibiotics disks used (BioRad, Marne-la-Coquette, France) were as follows: amoxicillin (25 μg), amoxicillin/clavulanic acid (20 μg/10 μg), ticarcillin (75 μg), ticarcillin/clavulanic acid (75 μg/10 μg), piperacillin (75 μg), piperacillin/tazobactam (75 μg/10 μg), imipenem (10 μg), cefalotin (30 μg), cefoxitine (30 μg), cefpodoxime (30 μg), cefotaxime (30 μg), ceftazidime (30 μg), cefpirome (30 μg), cefepime (30 μg), moxalactam (30 μg), aztreonam (30 μg), gentamicin (10 UI), tobramycin (10 μg), netilmicin (30 μg), amikacin (30 μg), isepamicin (30 μg), nalidixic acid (30 μg), levofloxacin (30 μg), ofloxacin (5 μg), ciprofloxacin (5 μg), tetracycline (30 μg), fosfomycin (30 μg), chloramphenicol (30 μg) and trimethoprim/sulfamethoxazole (1.25 μg/23.75 μg).

### PFGE-RFLP (Pulsed-Field Gel Electrophoresis - RFLP)

Genomic DNA was prepared in agarose plugs as previously described [28] and digested at 37°C with 40 U of *Spe*I (New England Biolabs). *Spe*I fragments were separated by PFGE using a CHEF-DRII apparatus (Bio-Rad, Laboratories) in a 1% agarose gel in 0.5× Tris-Borate-EDTA buffer (TBE) at 150 V and at 10°C. Pulse ramps were 5 to 35 s for 35 h followed by 2 to 10 s for 10 h. Molecular weight marker was a concatemer of phage l (New England Biolabs). The strains were randomly distributed among the different gels. *Spe*I-digested DNAs from strains ADV48 and ADV90 were respectively loaded in the first and the last well on each gel in order to standardize the migration patterns. Fingerprinting profiles generated by PFGE were standardized with PhotoCapt^® ^software (Vilbert Lourmat). The automated band detection was visually checked. The profiles were scored for the presence or absence of DNA bands. Restriction fragment variability was determined by the Nei and Li distance method modified by using the RESTDIST program in the Phylip package v.3.66 [29]. Clustering was predicated by the unweighted pair group average method (UPGMA) using the SplitsTree v4.0 [30,31].

### Gene amplification and sequencing

Genomic DNA was obtained using the Aquapure DNA extraction kit (EpiCentre). Seven genes (*dnaK*, *recA*, *rpoB*, *trpE, aroC, omp25 *and *gap*) were amplified using the primers shown in Table 3. PCR was carried out in 50 μL of reaction mixture containing 200 nM (each) primer (Sigma Genosys), 200 μM (each) desoxy-nucleoside triphosphates (dNTP) (Euromedex), 2.5 U of *Taq *DNA polymerase (Promega) in the appropriate reaction buffer and 50 ng of genomic DNA as the template. Amplification conditions were as follows: initial denaturation of 3 min at 95°C followed by 35-cycles with 1 min at 94°C, 1 min at 60°C (for *dna*K, *rpo*B *rec*A and *gap *fragments) or 1 min at 65°C (for *trp*E, *aro*C and *omp*25 fragments) and 2 min 30 s at 72°C. The final extension was carried out at 72°C during 10 min. PCR products and molecular weight marker (phage phiX DNA digested with *Hae*III, New England Biolabs) were separated in 1.5% (w/v) agarose gel in 0.5× TBE buffer. Amplification products were sequenced in both direction using forward and reverse sequencing primers (Table 3) on an ABI 3730xl automatic sequencer (Cogenics, France). The sequences were deposited to GenBank database with accession numbers: GQ429327 to GQ429816.

**Table 3:** Primers used for genes amplification and sequencing.

Locus	Putative gene Product	Locus position*	Gene size (bp)	Sequence length (bp)	Primers**	Primer sequence 5'-3'
*aroC*	Chorismate synthase	568275	1094	433	**43f**	TGGGGCGAAAGCCACGGTCTG
					
					**740r**	CCTTCACGGCGTTGATCGACA

*dnaK*	Heat shock protein 70 kDa	818851	1910	534	**591f**	CACGCTCGCCTGGAAGACGC
					
					1865r	GGGAACGACCAACTCCTGCGT
					
					**1777r**	TCGCTTACGGTCTGGACAAG

*gap*	Glyceraldehyde-3- phosphate dehydrogenase	1259699	1007	578	**138f**	TCTGCGTTATGACAGCGTTC
					
					940r	AAGCCCATTCATTGTCGTA
					
					**866r**	GAAGACCGAGGAATGGGAGT

*omp25*	25 kDa outer membrane protein	2714010	641	390	**188f**	ACGCGGAACTTGCTTTCGTCG
					
					**649r**	GCGCACTCTTAAGTCTCTCG

*recA*	Recombinase A	2079528	1085	490	**1f**	ATGTCTCAGAATTCATTGCGA
					
					988r	CTGACGAAGCGTGGTTTCGAT
					
					**697r**	ATACGGCGAATATCGAGACG

*rpoB*	Beta sub-unit RNA Polymerase	2046339	4133	501	**457f**	ATCGTTTCGCAGATGCACCG
					
					1449r	GACATACGTTCCTTGATCGCG
					
					**1119r**	TGACGCGATAGATGTCGAAC

*trpE*	Anthranilate synthase	1671911	2195	564	**15f**	TGCGGATAGCGAGATATTCCA
					
					1486r	GCCGATGCCTTCAATTCGGT
					
					**659r**	GTTGCCGTGCGAGACCAT

(f) forward primer; (r) reverse primer. Primers in bold were used for gene sequencing in both directions. (*) gene start codon position on the chromosome I sequence of *O. anthropi *ATCC 49188^T ^(accession number: CP000758). (**) primer denomination corresponded to its hybridization region in the gene according to the complete genome sequence of *O. anthropi *ATCC 49188^T^

### Phylogenetic analysis

Gene sequences were codon-aligned using ClustalX after translation with TRANSLATE http://www.expasy.org. The size of the codon-aligned sequences used for further analyses is indicated in Table 3. For phylogenetic analysis, concatenated sequences were re-aligned using ClustalW. Evolutionary distance was analyzed using Phylip package v3.66 [29] by Neighbor-Joining after distance matrix construction using DNADIST (F84 as substitution model). Bootstrap values were calculated using SEQBOOT and CONSENSE after 1000 reiterations. For Maximum likelihood (ML), the most appropriate substitution model determined according to Akaike information criterion calculated with Modeltest (v3.7) [32] was GTR plus gamma distribution and invariant sites. When gamma shape parameters were estimated from the dataset, ML phylogenetic analysis was performed using PHYML v2.4.6 [33]. ML bootstrap support was computed using PhyML after 100 reiterations. The sequence of *Brucella suis *1330^T ^was included in each phylogenetic analysis in order to place an artificial tree root.

### Multi Locus Sequence Typing (MLST)

The alignment obtained for phylogenetic treeing was used for assigning the isolates to a sequence type (ST) number according to their allelic profiles with the help of the non-redundant databases program http://linux.mlst.net/nrdb/nrdb.htm. A Minimum Spanning (MS) tree was constructed using Prim's algorithm to determine the links among STs http://www.pubmlst.org. MS clonal complexes included STs that differed by 2 or less alleles. Allele profiles were analysed using eBURST v3 software [34] to determine clonal complexes defined as sets of related strains that share at least five identical alleles at the 7 loci. MS clonal complexes were named MSCC followed by the ST number of the central ST in the tree. eBurst clonal complexes were named eBCC followed by the number of the predicted founder ST. When the founder is unpredicted or when the complex contained only 2 STs, the complex was named by the most represented ST or by default by the ST with the lower numbering. In both MS and eBURST analyses, the singleton (S) STs corresponded to STs differing from every other ST at 3 or more of the 7 loci. A distance matrix in nexus format was generated from the set of allelic profiles and then used for decomposition analyses with SplitsTree 4.0 software [30]. Program LIAN 3.1 [35] was used to calculate the standardized I_A _(sI_A_) and to test the null hypothesis of linkage disequilibrium as well as to determine mean genetic diversity (H) and genetic diversity at each locus (h). The number of synonymous (dS) and non-synonymous (dN) substitutions per site was determined on codon-aligned sequences using SNAP software [36].

## Results

### Development of a MLST scheme for *O. anthropi *typing

Since MLST approaches have never been performed for bacteria of the genus *Ochrobactrum*, we developed an original MLST scheme in this study. The choice of the seven loci was done on the basis of the complete genome sequence of *O. anthropi *ATCC 49188^T ^(accession number: CP000758). Amplification primers (Table 3) were designed using the alignment of genes from *O. anthropi *ATCC 49188^T ^and its closest totally sequenced relatives *Brucella suis *1330^T^, *Brucella melitensis *16M and *Brucella abortus *2308. We selected 6 genes encoding housekeeping products involved in transcription (*rpoB*), DNA repair (*recA*), stress response (*dnaK*), amino-acid biosynthesis (*aroC *and *trpE*) and the glycolytic pathway (*gap*) (Table 3). They were frequently used in MLST because mutations occurred slowly and were believed to be mostly neutral [37]. The seventh gene, *omp25*, encoding an outer membrane protein, was supposed to be a more variable marker. The selected loci were distributed as much as possible across the large chromosome of the bipartite genome of *O. anthropi *to ensure the absence of physical links between loci (Table 3). The MLST scheme showed between 4.5% to 13.7% of polymorphic sites among genes and a total of 235 single nucleotide polymorphisms (SNPs) in the 7 loci (Table 4). The mean genetic diversity (H) among strains was 0.7083 +/- 0.0506 and the genetic diversity at each locus (h) is given in Table 4. H in the clinical strains population (0.5959 +/- 0.0572) did not differ significantly from H in the environmental population (0.7301 +/- 0.0286), p = 0.11.

**Table 4:** Sequence analysis of the seven loci.

Locus	Number of alleles	Number of polymorphic sites (%)	Genetic diversity (h)	Number of non-synonymous codon	**d**_**N**_	**d**_**S**_	**d**_**N**_/**d**_**S**_
*dnaK*	6	24 (4.5%)	0.6625	3	0.0037	0.0811	0.0456
*recA*	6	32 (6.5%)	0.4286	0	0.000	-	-
*rpoB*	12	38 (7.6%)	0.7648	4	0.0036	0.1038	0.0346
*aroC*	15	59 (13.7%)	0.7478	5	0.0049	0.3239	0.0151
*omp25*	14	26 (6.6%)	0.8327	7	0.0044	0.0336	0.1309
*trpE*	14	58 (10.2%)	0.7892	9	0.0054	0.1417	0.0381
*gap*	12	35 (6.0%)	0.7321	2	0.0023	0.0926	0.0248

d_N _= non-synonymous substitutions per non-synonymous site. d_S _= synonymous substitutions per synonymous site

All gene fragments had equivalent mol% G+C contents from 56.7% to 61.4% with a mean value of 58.9% that was similar to the mean mol% G+C contents of the *O. anthropi *chromosomes (56.1%). The genes involved in amino-acid biosynthesis (*aroC *and *trpE*) appeared the most polymorphic. The gene *omp25 *that codes for an antigenic surface protein displayed a relatively low level of polymorphic sites (6.6%) but the highest genetic diversity level (0.8327). The majority of SNPs in all loci were synonymous (Table 4). However, the *omp25 *locus displayed the higher rate of non-synonymous SNPs versus synonymous SNPs. The non-synonymous mutations did not correspond to any premature stop codon.

### MLST revealed a human-associated clonal complex

The MLST data set for the 70 strains contained 44 genotypes or sequences types (STs) (Tables 1 and 2). The largest ST were ST1, ST3, ST4, ST5 and ST32, which contained 7, 6, 6, 3 and 4 isolates, respectively. All the strains belonging to ST3, ST4 and ST5 were clinical isolates whereas ST1 and ST32 grouped strains from man and environment. ST21, ST27 and ST35 corresponded to pairs of geographically unrelated environmental strains, ST7 and ST15 to pairs of clinical strains and the remaining 34 STs corresponded to clinical (n = 22) and environmental (n = 12) unique strains. The number of STs per strain did not vary between the clinical (0.64) and the environmental population (0.61).

**Figure 1:**
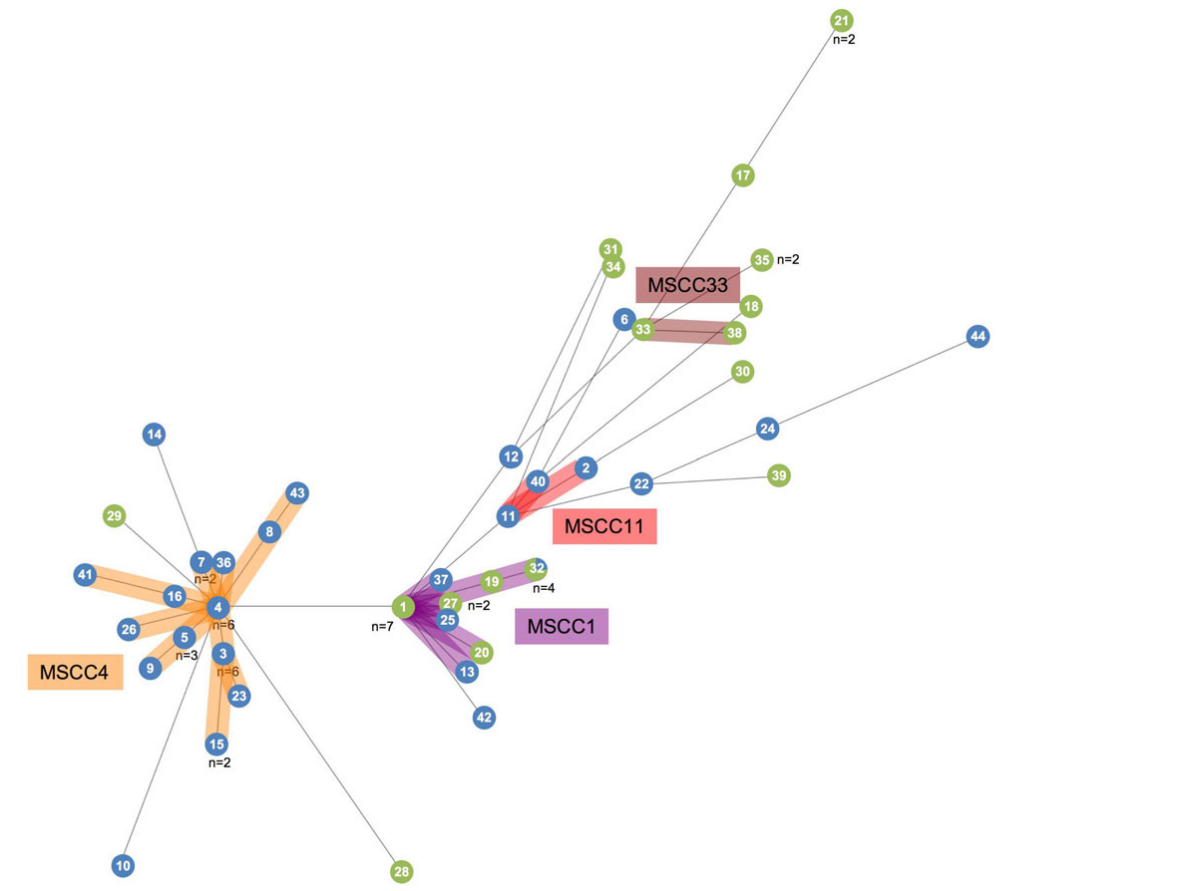
**Minimum-spanning tree based on MLST data**. Colours indicate the source (clinical in blue or environmental in green) of the strains. The number given in the circle corresponds to the sequence type (ST) number. The number given near the circle corresponds to the number of isolates presenting the ST. The size of circles is proportional to the number of isolates representing the ST. MSCC for Minimum Spanning Clonal Clomplex.

We constructed a minimum-spanning (MS) tree based on clustering of the MLST profiles as a graphic representation of the population structure (Fig. 1, Tables 1 and 2). In the MS tree, strains formed two major MS clonal complexes MSCC1 (19 strains of both human and environmental origin, 9 STs) and MSCC4 (27 human strains, 13 STs) as well as two minor complexes, MSCC11 (3 human strains, 3 STs) and MSCC33 (2 environmental strains, 2 STs). Using eBURST software [34], the 44 STs were divided into 2 major clonal complexes, eBCC1 (23 strains of both human and environmental origin; 13 STs; ST1 as predicted founder) and eBCC4 (27 human strains; 13 STs; ST4 as predicted founder), 3 minor clonal complexes eBCC31, eBCC21 and eBCC35 each including 3 strains and 11 singleton STs (Tables 1 and 2).

MSCC4 and eBCC4 grouped the same strains and STs (Table 1). MSCC1 grouped 18 strains out of the 23 associated to eBCC1. By MS analysis, the five remaining STs grouped in eBCC1 belonged to MSCC11 (3 human strains; ST2, ST11, ST40) or were singleton STs (ST12, ST29). Other incongruence was observed between minor clonal complexes detected by eBURST and MS treeing. eBCC21 and eBCC35 were split in singleton STs in the MS tree. MSCC33 grouped 2 strains out of the 3 forming eBCC31.

Most of the human clinical isolates (26/43) belonged to MSCC4/eBCC4 that exclusively contained human strains (Table 1; Fig. 1). The type strain of *O. anthropi*, for which the human clinical origin is highly probable albeit unproved [38], also belonged to this complex. The 17 other clinical strains were scattered in MSCC1/eBCC1 beside environmental strains or corresponded to MSCC11 or to singleton STs.

The strains belonging to MSCC4/eBCC4 colonized or infected diverse clinical sites. They were isolated in France (different distant hospitals), Denmark, Sweden, United Kingdom and USA between 1971 and 2007, suggesting that their clustering in the same complex did not reflect cross contamination or spread among a restricted population of patients. Of note, strains isolated at the same period and in the same hospital could belong to different STs and complexes (Tables 1 and 2). For instance, the strains ADV88, ADV90 and ADV91 isolated from the digestive tract of patients hospitalized in Montpellier (France) in May 2007 belonged to different clonal complexes or to singletons. Moreover, the strains CLF18, CLF19 and CLF20 were isolated in throat samples of the same patient but presented different STs.

No differences were observed regarding geographic origin, clinical site isolation or clinical situation between MSCC4/eBCC4 strains and other human strains.

Among environmental isolates, no relationships between STs or complexes and habitats, geographic origins or year of isolation could be established (Tables 2). For instance, the 6 strains isolated in association with *Photorhabdus luminescens *from the nematode *Heterorhabditis indica*, including two Italian strains (2006) and two Guadeloupian strains (1996), belonged to diverse STs and/or complexes. Conversely, MSCC1 grouped a strain isolated in 2006 in Argentina and a strain from Sweden isolated in 1978. The reference strain of the species *O. lupini *shared its ST, ST35, with a strain of *O. anthropi *isolated in a denitrification reactor. *O. cytisi *was represented by a singleton ST.

Finally, the structure of the population tested herein, particularly the existence of a human-associated clonal complex (MSCC4/eBCC4) suggested difference in the propensity of *O. anthropi *to live in association with human beings.

### Multi-locus sequence-based phylogeny

**Figure 2:**
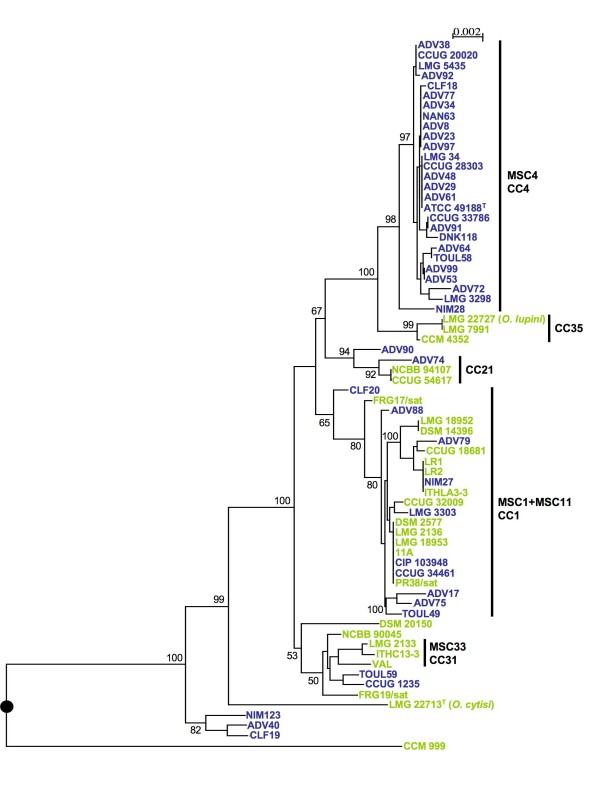
**ML trees based on concatenated sequences of the seven housekeeping gene fragments**. Position of the artificial root (black circle) corresponded to branching of the out-group (*B. suis *1330^T^) included in the analysis but not shown on the tree. Horizontal lines are scales for genetic distance. Numbers given at the nodes are support values estimated with 100 bootstrap replicates. Only bootstrap values >50% are indicated. For better visualization of the tree, bootstrap values are shown at the terminal nodes. The scale bar indicates the number of substitutions per nucleotide position. The clonal complexes MSCC and eBCC determined by Minimum Spanning and eBurst, respectively are indicated by vertical bold bars. Blue: clinical strains; green: environmental strains. (*) indicated major conflicting phylogenetic positions between the seven genes-based tree and the *trpE*-based tree in Fig 3.

We applied distance and ML phylogenetic approaches to the concatenated sequences (3490 nucleotides) of the seven loci from all STs. The two methods gave congruent trees and the ML tree is presented in Fig. 2. The topology of the trees confirmed the population structure determined by MS treeing and eBURST. A large and robust clade grouped 27 strains from human origin and corresponded to the major clonal complex MSCC4/eBCC4. The clade corresponding to eBCC1 contained 23 strains from different origins. In this clade, the relationships between environmental and clinical strains could not be established due to the weak robustness of the branching order.

**Figure 3:**
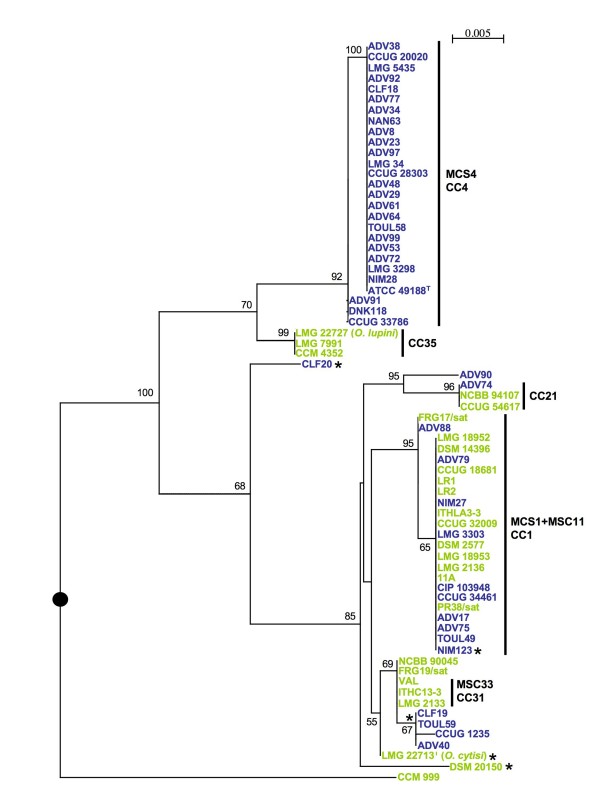
**ML trees based on the *trpE *gene fragment**. Position of the artificial root (black circle) corresponded to branching of the out-group (*B. suis *1330^T^) included in the analysis but not shown on the tree. Horizontal lines are scales for genetic distance. Numbers given at the nodes are support values estimated with 100 bootstrap replicates. Only bootstrap values >50% are indicated. For better visualization of the tree, bootstrap values are shown at the terminal nodes. The scale bar indicates the number of substitutions per nucleotide position. The clonal complexes MSCC and eBCC determined by Minimum Spanning and eBurst, respectively are indicated by vertical bold bars. Blue: clinical strains; green: environmental strains. (*) indicated major conflicting phylogenetic positions between the seven genes-based tree (Fig. 2) and the *trpE*-based tree.

The sequences of each of the seven loci were used in the ML analysis of congruence where each ML tree was compared to the ML tree reconstructed from the seven concatenated sequences. We observed conflicting topologies regarding the tree based on concatenated sequences suggesting recombination events, particularly for the *aroC*- and *omp25*-based trees (data not shown). The *dnak*-, *recA*- and *rpoB*-based trees were more congruent. They affiliated the isolates to only 2 to 3 large clades but they failed to establish relationships inside the clades. However, the combination of the 3 markers gave a tree showing polymorphism inside each clade. Particularly, the strains belonging to eBCC1 and MSCC4/eBCC4 formed two independent robust lineages (data not shown). The *gap*- and *trpE*-based trees were globally congruent with the tree based on concatenated sequences. The gene *trpE *appeared to be a good marker for studying the phylogenetic relationships among isolates in the species *O. anthropi *(Fig. 3).

Strain CCM 999 generally branched out of the other strains of *O. anthropi *suggesting that this strain could belong to another *Ochrobactrum *species. The phylogenetic positions of the clinical strains CLF19 and ADV40 significantly varied according the markers, suggesting important recombination events. For instance, in the *aroC*-based tree, CLF19, ADV40, NIM123 and the atypical strain CCM 999 grouped together since the four strains shared exactly the same *aroC *locus. The position of *O. cytisi *LMG 22713^T ^varied according to the marker, an external position to *O. anthropi *was only observed in *aroC*, *rpoB *and *omp25-*based trees. *O. lupini *LMG 22727 with two environmental *O. anthropi *strains formed a clade branching inside *O. anthropi *in all trees (Fig 2 and 3).

### Recombination in *Ochrobactrum anthropi*

**Figure 4:**
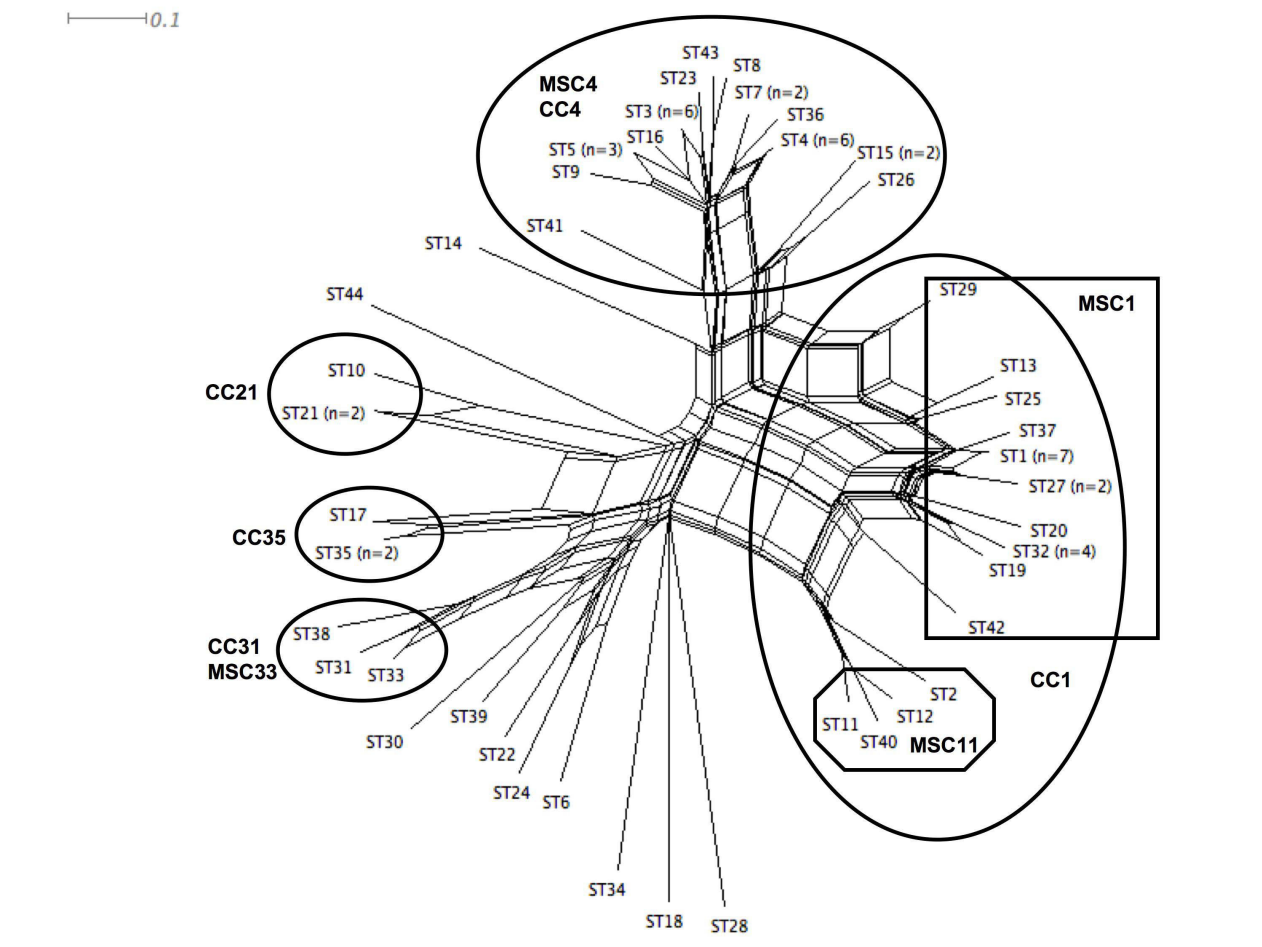
**SplitsTree decomposition analyses of MLST data for *O. anthropi *strains**. The distance matrix was obtained from allelic profiles of strains. The clonal complexes (MSCC and eBCC) are delineated by bold lines.

We assessed the linkage between alleles from the 7 loci by determination of sI_A _value. sI_A _value is expected to be zero when a population is at linkage equilibrium, i.e., that free recombination occurs. Analyses were carried out using either all isolates or all STs (i.e. one isolate from each ST) in order to minimize a bias due to a possible epidemic population structure. sI_A _was significantly different from zero when all isolates were included in the analysis (sI_A _= 0.3447; p = 0.0041) or when only one isolate from each ST was included (sI_A _= 0.2402; p = 0.0031). The population studied displayed linkage disequilibrium suggesting a low rate of recombination. However, linkage disequilibrium could be present into long-term recombining populations where adaptative clones emerge over the short-term [39]. To explore this hypothesis, we performed decomposition analysis that depicts all the shortest pathways linking sequences, including those that produce an interconnected network [30]. A network-like graph indicates recombination events. The split graph (NeighborNet) of all seven loci displayed a network-like structure, with parallel paths. However, the network generated clusters consistent with MLST major clonal complexes and phylogenetic lineages (Fig. 4). Recombination events appeared more frequently inside each major and minor clonal complex. *O. cytisi *LMG 22713^T ^as well as strains CCM 999, DSM 20150 and ADV90 corresponding to singleton STs, ST34, ST18, ST28 and ST14, respectively, were less subject to recombination events with other strains. On the contrary, the strains in singleton STs ADV40 (ST6), CLF19 (ST24), FRG19/sat (ST30), CCUG1235 (ST22), TOUL59 (ST44) and NCCB 90045 (ST39) were suspect to recombination (Fig. 4). The positions of these strains in the phylogenetic trees varied according to the markers, as shown before and in Fig. 2 and 3.

### High diversity of PFGE genomotypes

**Figure 5:**
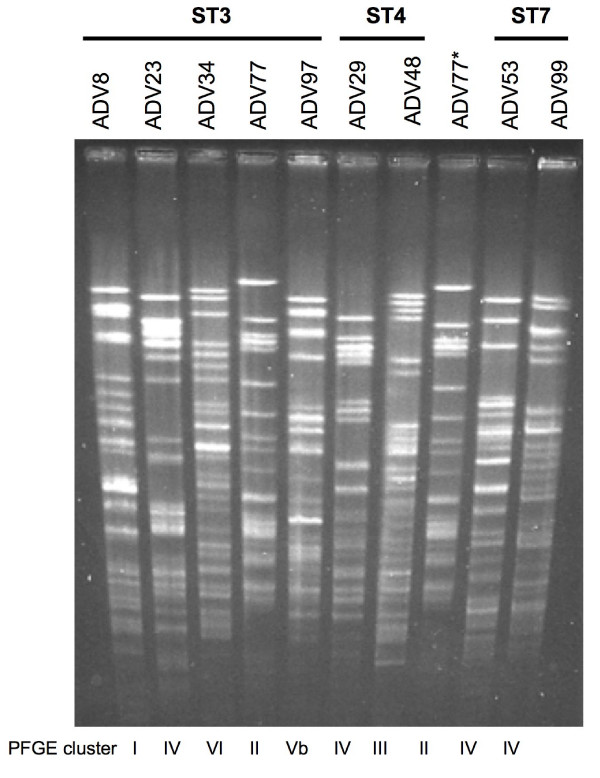
**Representative PFGE profiles obtained for French clinical strains isolated in the same hospital and belonging to the major clonal complex MSCC4/eBCC4**. PFGE clusters at a 60% similarity level are indicated at the bottom of the gel. (*) ADN of the strain ADV77 was deposited twice on the gel to check reproducibility and to help profiles comparison.

The genomic DNA of 56 *O. anthropi *strains (32 human and 24 environmental) were analysed by PFGE. At a 100% similarity level, PFGE discriminated all the strains except LR1 and LR2, which came from the same environmental sample. The pulsotypes were highly diverse even among strains belonging to the same clonal complex and/or sharing the same ST. The clinical strains originating from a same French hospital were epidemiologically unrelated by PFGE analysis (Fig. 5). PFGE clusters appeared only below a 60% similarity level (Tables 1 and 2), suggesting that PFGE was unable to structure the population studied. Members of the different clonal complexes appeared intermingled among the PFGE clusters (Tables 1 and 2). The PFGE clusters defined at 60% similarity level could not be related to any characteristic of the strains such as isolation niche, geography, lifestyle, date of isolation, or antibiotype.

### Antibiotypes of *O. anthropi *clinical and environmental strains

Both clinical and environmental strains appeared highly resistant to all β-lactams, but imipenem. We observed a general susceptibility to aminoglycosides, fluoroquinolones, tetracycline, trimethoprim-sulfamethoxazole and an overall resistance to chloramphenicol and fosfomycin. The strains isolated from hospitalized patients did not show particular resistance characteristics when compared to environmental strains. This suggested that the high level of resistance observed in *O. anthropi *is a natural trait of the species mostly unrelated to the medical use of antibiotics.

## Discussion

We proposed here the first application of MLST to *O. anthropi*. Our MLST scheme contains 6 housekeeping and 1 outer-membrane protein (*omp25*) genes, scattered on the large chromosome of strain ATCC 49188^T^. The sequences of bipartite genomes in alphaproteobacteria suggested the plasmidic origin of the smaller chromosome [40]. In this MLST scheme, no loci were chosen on the small chromosome to avoid bias due to the potential difference in the evolution history of the two chromosomes. The construction of another complete MLST scheme based on genes carried by this second chromosome would be of great interest to assess the emergence and the evolution of the complex genome in *O. anthropi*.

At each locus examined by MLST, even at *omp25*, genetic variation appears to be mostly neutral. The 7 loci had mol%G+C contents similar to that of the rest of the genome. This suggests that these genes were not recently acquired through horizontal gene transfer. ST diversity in *O. anthropi *appeared similar to that of a significant number of bacteria (0.63 ST per isolate); see [37] for a review. This level of STs diversity allowed a wide range of applications from strain characterisation to population structure analysis and to evolutionary studies [37]. A MLST scheme has been recently proposed for *Brucella *spp., the genus phylogenetically most related to *Ochrobactrum *[41]. The genes *dnaK*, *gap*, *omp25 *and *trpE *were analysed for both *Brucella *spp. and *O. anthropi*. Considering these 4 loci, genetic diversity in *O. anthropi *(6.6 polymorphic nucleotides per 100) appeared 5-fold higher than observed in the genus *Brucella *(1.4%). This difference in genetic diversity could reflect differences in lifestyles, qualifying *O. anthropi *as a versatile generalist and *Brucella *as a narrow niche-specialist. The *recA *gene displayed the lower genetic diversity in our scheme. It was previously used for studying the phylogenetic interrelationships among members of the family *Brucellaceae *and appeared also unable to distinguish between some species in the genus *Ochrobactrum *[9]. We confirm here the high conservation of this marker and its inefficiency to explore the interrelationships in the species *O. anthropi*. The *rpoB *and *dnaK *sequences were also conserved among strains of *O. anthropi*. These results justified multi-locus approaches rather than single target-based analyses for sub-typing *O. anthropi*. However, in our MLST study, two markers reflected the overall diversity determined by the 7 loci. This was the case for *trpE *and to a lesser extent for the *gap *gene. Differing from *rrs *and *recA, trpE *and *gap *were less conserved and gave a tree with robust phylogenetic interrelationships at the sub-species level. These two markers could be tested at the intra- and the inter-genus level in order to solve conflicting taxonomic positions in the family *Brucellaceae *[9].

The population of 70 strains of *O. anthropi *appeared structured in 2 major and 3 minor clonal complexes. The calculation of standardized I_A _indicated a linkage disequilibrium that also evoked a clonal population structure. However, split decomposition analysis resulted in a network-like graph indicating a significant level of recombination mostly inside clonal complexes. Moreover, phylogenetic conflicts were observed when the trees based on different markers were compared. The persistence of a linkage disequilibrium in populations in which recombination is frequent could be due to an epidemic population structure or to a mix of ecologically separated subpopulations [39]. Our results were compatible with an epidemic population structure composed of a limited number of clones originating from a background of unrelated genotypes recombining frequently. Our results were also compatible with a mix of ecologically separated populations i.e. environmental and clinical strains. These two hypotheses fitted with the existence of a human-associated subpopulation that either emerged as an epidemic clonal complex or encountered limited genetic exchanges with other populations. Testing a larger collection of strains from diverse origins could address this question. Diverse methods have been proposed for the molecular typing of bacteria in the genus *Ochrobactrum*. ITS1 sequencing and rep-PCR have been successfully used to assess the level of microdiversity in the genus as well as to cluster the strains according to the species [12,13]. However, within the species *O. anthropi *there was no correlation between rep- or ITS1-based clusters and origin of the strains. In the collection tested, MLST data and multi-locus-based phylogeny provided evidence of a clonal complex associated to human beings.

To strengthen this evidence, the question of the representativeness of the human strains included in the MLST analysis should be addressed. Most clinical strains originated from France (n = 34) but they have been isolated in diverse regions and at different times from 1998 to 2007. We also included 9 geographically unrelated clinical strains isolated in Scandinavia, United Kingdom or Louisiana (USA) from 1971 to 1995. Seven of them belonged to the major complex MSCC4/eBCC4 beside most of the French clinical isolates. This indicated that MSCC4/eBCC4 could be considered as a human-adapted subpopulation rather than a geographic subpopulation. The mean genetic diversity calculated from the seven loci showed no significant differences between clinical isolates and isolates from all other various origins. This is also the case for the number of STs per strain. The genetic diversity of the clinical population was confirmed at the genomic level since all the clinical strains displayed different pulsotypes indicating that they were epidemiologically unrelated. Therefore, epidemiological, genetic and genomic data exclude a bias in strain sampling and enhance the robustness of the human-associated subpopulation described herein.

PFGE typing appeared highly discriminative in the species *O. anthropi *since only 2 strains originating from the same environmental sample displayed the same pulsotype. None of the isolates originating from one hospital displayed the same pulsotype. This wide genomotype diversity observed here confirmed previous data showing the genomic plasticity of *O. anthropi *[28]. Genomic rearrangements in plastic genomes are considered as rapid evolution mechanisms, named micro-evolution with respect to the time-scale, that could be involved in rapid adaptation processes to a particular niche [42]. Restriction fragment length polymorphism in PFGE detected genomic modifications such as rearrangements and horizontal genetic transfer events rather than single nucleotide polymorphisms [43]. The higher discriminative power of PFGE suggested that large rearrangements occurred at higher rates than intragenic point mutations in housekeeping genes in *O. anthropi*. Despite its discriminative ability, genomotyping failed to structure the bacterial population with respect to the habitat or the origin of the strains, probably due to the lack of close relationships among the strains. The same results were obtained in previous studies based on rep-PCR where clinical, soil and rhizosphere isolates of *O. anthropi *appeared intermingled in a defined genomotype [13,15]. Finally, genomotyping methods appeared to be the most suitable to identify a particular *O. anthropi *clone but should be applied to cross-contamination or to outbreak tracing rather than to population structure assessment.

The emergence of clinical-encountered subpopulations could be caused by the acquisition of genes involved in antimicrobial resistance that conferred a strong selective advantage in the hospital environment. In the case of *O. anthropi*, we observed no differences in antimicrobial resistance patterns between hospital-acquired and environmental strains. Moreover, most of the genes analysed were not affected by the antibiotic selective pressure. The *rpoB *gene could be object of Darwinian selection by antibiotics since RNA polymerase is the target for rifampicin. This is also the case for the *omp25 *gene that could be involved in the resistance to a range of antibiotics. However, dN/dS showed that *rpoB *and *omp25 *modifications corresponded to neutral rather than to Darwinian-selected mutations in the population studied. Therefore, resistance to antimicrobial agents could not explain the selection of the human-associated complex MSCC4/eBCC4 in the population of *O. anthropi *studied here. Beside, even if the apparition of MSCC4/eBCC4 clonal complex was not dated, one can hypothesize from the slow evolution rate of the investigated genes that it probably emerged a long time ago before being submitted to antibiotic pressure.

The existence of human-associated subpopulation unrelated to antibiotic selective pressure, in a natural population of *O. anthropi*, suggested that a subpopulation of this bacterium could be considered as "specialized opportunistic" pathogen. In the case of *Pseudomonas aeruginosa*, another versatile bacterium, the clinical isolates are not specialists since *P. aeruginosa *environmental isolates are indistinguishable from clinical isolates [44]. The same situation was observed here for *O. anthropi *grouped in the clonal complex eBCC1. One could consider that the virulence traits of *P. aeruginosa *reflect characters acquired by the species to survive in the environment. Analysis of the complete genome sequence of *O. anthropi *showed a complete *virB *operon, which codes for a putative type IV secretion system known to be the major virulence factor in *Brucella *spp. and in *Agrobacterium tumefaciens*, two phylogenetic neighbours of *Ochrobactrum *spp. [23]. Analysis of *virB *polymorphism in the *O. anthropi *population will be of great interest. However, *O. anthropi *is a mild pathogen that generally causes diseases in immunocompromised patients. It probably does not display typical virulence factors but rather "human-adaptation" traits. These traits might be non-equally distributed in the population and could explain the emergence of human-adapted lineages. The detection of a human-specialized lineage in our collection of *O. anthropi *suggests that this versatile bacterium could be a good model to better understand the emergence of phylogenetically related strict pathogens of animals and plants, such as *Brucella*, *Bartonella *and *Agrobacterium*.

## Conclusion

We confirmed the high discriminative power of PFGE for subtyping *O. anthropi*. However, this method failed to structure the population and should be reserved to investigation of epidemiologically closely related strains. The MLST scheme gave preliminary results, which could be emended after enrichment of the STs database. For this purpose, the MLST scheme and data will be deposited to the website MLST http://www.mlst.net. MLST on *O. anthropi *allowed for the first time (1) to identify a human-specialized subpopulation, (2) to show an epidemic population structure, (3) to evaluate the recombination rate. Moreover, we showed that our MLST scheme could be useful for a taxonomic purpose in order to clarify systematics in the *Brucellaceae*.

Evidence of a human-associated clonal complex suggested a specialized opportunistic behaviour for *O. anthropi*. This study underlines the interest of studying the housekeeping genetic background in opportunistic pathogens, for which specific virulence traits remain unknown.

## Authors' contributions

SR carried out the molecular genetic and genomic studies, participated in the sequence alignment, phylogeny and manuscript draft. FA participated in the MLST design and analyses, carried out complementary molecular genetic assays, sequence alignments and sequence quality checking. EJB conceived of the study and coordinated it, performed MLST data analysis and drafted the manuscript. AM is the curator of the clinical isolates collection. JLJ designed and carried out antimicrobial susceptibility testing. EF provided clinical isolates and critically read the manuscript. HM participated in the design of the study, in the characterisation of clinical isolates and helped to draft the manuscript. CT participated in the study design, coordinated PFGE and phenotypic studies, participated in data analysis and helped to draft the manuscript. All authors read and approved the final manuscript.
